# Association between burnout and stigma in physicians

**DOI:** 10.1371/journal.pone.0283556

**Published:** 2023-04-05

**Authors:** S. Favre, N. M. Bajwa, M. Dominicé Dao, M.-C. Audétat Voirol, M. Nendaz, N. Junod Perron, T. Perneger, H. Richard-Lepouriel

**Affiliations:** 1 Mood Disorder Unit, Psychiatric Specialties Service, Geneva University Hospital, Geneva, Switzerland; 2 University of Geneva Faculty of Medicine, Department of Paediatrics, Unit of Development and Research in Medical Education (UDREM), Geneva University Hospital, Geneva, Switzerland; 3 Division of Primary Care Medicine, Geneva University Hospital, Geneva, Switzerland; 4 Faculty of Medicine, Institute of Primary Care (IuMFE) University of Geneva, Unit of Development and Research in Medical Education (UDREM), Geneva, Switzerland; 5 University of Geneva Faculty of Medicine, Division of General Internal Medicine, Unit of Development and Research in Medical Education (UDREM), Geneva University Hospital, Geneva, Switzerland; 6 Institute of Primary Care, Geneva University Hospital, Geneva, Switzerland; 7 University of Geneva Faculty of Medicine, Unit of Development and Research in Medical Education (UDREM), Geneva, Switzerland; 8 Division of Clinical Epidemiology Division, Geneva University Hospital, Geneva, Switzerland; 9 University of Geneva Faculty of Medicine, Department of Psychiatry, Geneva, Switzerland; Universidad de Zaragoza, SPAIN

## Abstract

**Background:**

Physicians suffering from burnout are more likely to develop depression, substance dependence, and cardiovascular diseases, which can affect their practices. Stigmatization is a barrier to seeking treatment. This study aimed to understand the complex links between burnout among medical doctors and the perceived stigma.

**Methods and findings:**

Online questionnaires were sent to medical doctors working in five different departments of the Geneva University Hospital. The Maslach Burnout Inventory (MBI) was used to assess burnout. The Stigma of Occupational Stress Scale in Doctors (SOSS-D) was used to measure the three stigma dimensions. Three hundred and eight physicians participated in the survey (response rate: 34%). Physicians with burnout (47%) were more likely to hold stigmatized views. Emotional exhaustion was moderately correlated with perceived structural stigma (r = 0.37, P < .001) and weakly correlated with perceived stigma (r = 0.25, P = 0.011). Depersonalization was weakly correlated with personal stigma (r = 0.23, P = 0.04) and perceived other stigma (r = 0.25, P = 0.018).

**Conclusion:**

These results suggest the need to adjust for existing burnout and stigma management. Further research needs to be conducted on how high burnout and stigmatization impact collective burnout, stigmatization, and treatment delay.

## 1. Introduction

Physician burnout is widely defined as emotional exhaustion, a decreased sense of meaning at work, feelings of ineffectiveness, and the dehumanization of others [1,2]. Burnout is an important issue among medical doctors, with a prevalence of 25–65% [3–5]. The Physicians Foundation (USA) (2020 survey) reported that 58% of physicians often have feelings of burnout, compared to 40% in 2018. Burnout among physicians may lead to decreased empathy and attention toward patients [6,7], the decline in mental health among doctors, depression, substance abuse, suicide [8,9], and a higher risk of cardiovascular disease [10]. “The burned-out physician” is described as “angry, irritable, impatient” and “having increased absenteeism, decreased productivity, and providing decreased quality of care” [11]. Burnout can impact quality of care by poor medical knowledge [12], less thorough therapeutic discussions, less adequate or even erroneous prescriptions, particularly among internal medicine residents [13–15]. Burnout in medical doctors can represent a high cost, as it is also related to early departures [4,12] and an increased desire to change professions [16]. Medical turnover about job dissatisfaction increases the costs of recruiting and retaining doctors [12,17] and decreases cohesion in the institution [18].

Burnout is associated with environmental factors, such as a high prevalence of occupational stress among physicians [19–21]. Heavy schedules, working time, stress, and regular on-call duties are different factors that can promote burnout [22,23]. On a more general level, the healthcare system is often experienced as inequitable, focused on financial profitability, and dehumanized [24]. Pressure to lower costs and increase productivity can induce burnout among medical doctors, who are caught between profitability requirements and their contact with people suffering [25]. Publication pressure for doctors engaged in academic careers or poorly supervised technical acts have also been mentioned as risk factors [26]. On the personal level, the imbalance between work and private life can produce potential conflicts between professional and personal choices, usually solved in favor of professional life [27]. These difficulties can occur in fragile social realities, such as marital or financial difficulties, or in the isolation of doctors from abroad [10].

Stigma refers to negative social attitudes that are attached to a disapproved mark in an individual.

Link & Phelan [28] conceptualized the mechanisms of stigmatization. The first mechanism (“direct discrimination”) involved attitudes and beliefs producing direct discriminatory behaviors. The second mechanism (“structural discrimination”) involved structural inequalities producing covert discrimination. The third mechanism (“social psychological processes”) involved self-stigmatization producing self-devalued behaviors. These interrelated mechanisms can reciprocally increase, leading to detrimental consequences for the stigmatized individuals

Stigma is a barrier to help-seeking, and many physicians experiencing burnout will not be treated or experience delay in treatment [29–31]. In medical culture, burnout is highly stigmatized because physicians tend to consider their state of health, especially mental health, as an indicator of their medical competence. A doctor suffering from burnout may be perceived by their peers and by their selves as weak and incompetent [10,32]. Stigma has been defined as including different components: personal, perceived other, and perceived structural stigma [33]. Personal stigma refers to personal stigmatizing attitudes held toward other people experiencing occupational stress and burnout, perceived other stigma is defined as a person’s beliefs about the stigmatizing attitudes held by other people. Perceived structural stigma represents policies and practices that restrict the opportunities or well-being of the stigmatized person [33].

Nearchou et al. [34] reported the importance of separately investigating the different types of stigmas when predicting help-seeking interventions. They showed that public stigma was a stronger predictor of help-seeking intentions for depression in adolescents than personal stigma. Bracke et al. [35] also stressed the need to distinguish between the different types of stigmas. They reported that dominant beliefs about stigma within a culture decreased the likelihood of seeking help from mental health professionals compared to individuals’ personal beliefs regarding stigma. To improve help-seeking among physicians, the effects and interrelationships between these types of stigmas and burnout must be investigated. More profound knowledge of stigmatizing thoughts and erroneous beliefs is essential to reduce the barriers to treatment, improve the prevention and management of burnout in medical doctors, as well as increasing the quality of care. Addressing and challenging stigma may result in better outcomes for physicians and patients alike [36]. However, very little data are available concerning the stigma of burnout among medical doctors.

This study aimed to investigate the potential relationship between stigma and burnout symptomatology among medical doctors working in a university hospital and, more specifically, between the dimensions of stigma and burnout. We hypothesized that:1) personal stigma could be associated with emotional exhaustion as a defense or denial of one’s own emotional difficulties; 2) perceived other stigma could be related to depersonalization and development of negative interpersonal attitudes toward patients and/or colleagues, as beliefs about others’ stigmatizing attitudes and negative attitudes toward others can enhance each other in a loop; and 3) perceived structural stigma could be correlated with personal accomplishment, as perceived restriction and loss of confidence can interact in a vicious circle.

These hypotheses are issued from Link & Phelans’ discrimination model integrated with the Maslach model of burnout.

## 2. Methods

### 2.1 Participants and setting

This study was conducted in five academic departments at Geneva University Hospital (HUG). Psychiatry, internal medicine, family medicine, pediatrics, obstetrics, and gynecology. Nine hundred and sixty-one residents, fellows, and attending physicians were invited to complete an electronic survey via email. They received two reminders over three months. Participation in the study was voluntary and there was no compensation for completing the questionnaire. The study protocol was approved by the Research Ethics Committee of Geneva University Hospital. Written informed consent for the study was obtained from all participants. All survey responses were anonymous. After completion of the survey, mental health resources were provided to the participants if needed.

### 2.2 Measurement of burnout and stigma

Burnout was assessed using the *Maslach Burnout Inventory* (MBI) [37]. The French version of this 22-item self-report scale is answered on a 6-point Likert scale (from “never” to “every day”) [38]. The scale measures three dimensions: emotional exhaustion (feeling of emotional annihilation of resources and loss of motivation), depersonalization (development of negative interpersonal attitudes toward patients and/or colleagues with loss of empathy, irritability, and cynicism), and personal accomplishment (loss of confidence in one’s competence with the idea of being unable to achieve one’s work successfully. Higher scores on emotional exhaustion (≥ 27) and depersonalization (≥ 1) and low scores on personal accomplishment (≤33) are indicative of greater burnout [39].

Stigma was assessed using the *Stigma of Occupational Stress Scale for Doctors* (SOSS-D) [33]. It is an 11-item self-report scale that measures three dimensions of stigmatization: perceived structural stigma (addresses stigma of stress and burnout within the context of structural influences operating in the workplace, policies, and practices that restrict the opportunities or well-being of the stigmatized physician), personal stigma (personal stigmatizing attitudes held toward other people experiencing occupational stress and burnout), and perceived other stigma (addresses a physician’s beliefs about the stigmatizing attitudes held by other physicians). The scale was developed based on stigma-related barriers to help-seeking in the medical profession. It was meant to improve the understanding of stigma in this population and assist in designing strategies to enhance help-seeking among physicians. Responses are recorded on a 7-point Likert scale ranging from “strongly disagree” to “strongly agree.” In the French version of the scale, five items compose the “perceived structural stigma subscale” (3*, 5, 6, 8, 9*), and two of them (*) are intended to be reverse-scored. Three items compose the “personal stigma” subscale (1, 7, and 11), and three the “perceived other stigma” subscale (2, 4, and 10). The SOSS-D was translated into French (and back-translated) and piloted with 12 physicians. After the initial adjustments to the translation, the scale was administered to 323 physicians. The internal consistency coefficient for the French SOSS-D was 0.72 and ranged from 0.55 to 0.75 for the three subscales. Confirmatory factor analysis indicated an acceptable but imperfect fit of the hypothesized measurement model (root mean square error of approximation 0.89, comparative fit index 0.867). The results provide evidence of the validity and reliability of the French version of the SOSS-D.

### 2.3 Statistical analysis

Descriptive statistics were used to show the respective means of the different burnout and stigma subscales. The stigma scale was stratified by burnout status and mean comparison of both groups. Associations between the subscales were made using univariate associations that included all three MBI subscales as interdependent covariates, with all variables standardized to mean 0 and variance 1. Adjusted associations were obtained from multiple linear regression models that included all three MBI subscales as covariates, with all variables normalized to mean 0 and variance 1 (equivalent to partial correlation coefficients). Attenuation correction was performed by dividing each partial correlation coefficient by the square root of the product of the internal consistency coefficients of the corresponding scales. All analyses were conducted using Stata SE 16 for Macintosh [40].

## 3. Results

### 3.1 Participants

Three hundred and eight physicians participated in the survey (response rate: 34%). Sixty-two percent (62%) of the 308 physicians who completed the demographic portion of the survey were women. Most participants (77%) were in a personal relationship. The mean age was 38.6 years (SD = 9.2). Forty percent of the participants were residents, 36.8% were fellows, and 23% were attending physicians. They had practiced for an average of 12.5 (SD = 9.4) years. Internal medicine physicians represented 29% of the participants, followed by psychiatrists (23%), pediatricians (22%), family medicine physicians (21%), and obstetricians & gynecologists (5%).

### 3.2 Prevalence of burnout and stigma

The descriptive statistics are presented in Table 1.

**Table 1:** Descriptive Scale Statistics Maslach’s Burnout Inventory and SOSS-D.

	Mean (SD)	Interquartile Range	Cronbach’s alpha	Percentage meeting criteria for burnout low	Percentage meeting criteria for burnout moderate	Percentage meeting criteria for burnout high
**Maslach’s Burnout Inventory (N = 308)**						
**Emotional Exhaustion**	20.26 (11.46)	11;28	0.92	44.8%	35.5%	19.7%
**Depersonalization**	7.88 (6.32)	3;12	0.78	44.8%	28.7%	26.5%
**Personal Accomplishment**	39.33 (6.10)	36;44	0.75	54.8%	31.6%	13.6%
**SOSS-D (N = 308)**						
**Perceived Structural Stigma**	3.85 (.68)	3.5;4.25	0.65			
**Personal Stigma**	2.58 (1.18)	1.67;3.33	0.55			
**Perceived Other Stigma**	4.12 (.89)	3.5;4.75	0.66			

### 3.3 Perceived stigma in physicians stratified by the presence of burnout

**Fig 1:**
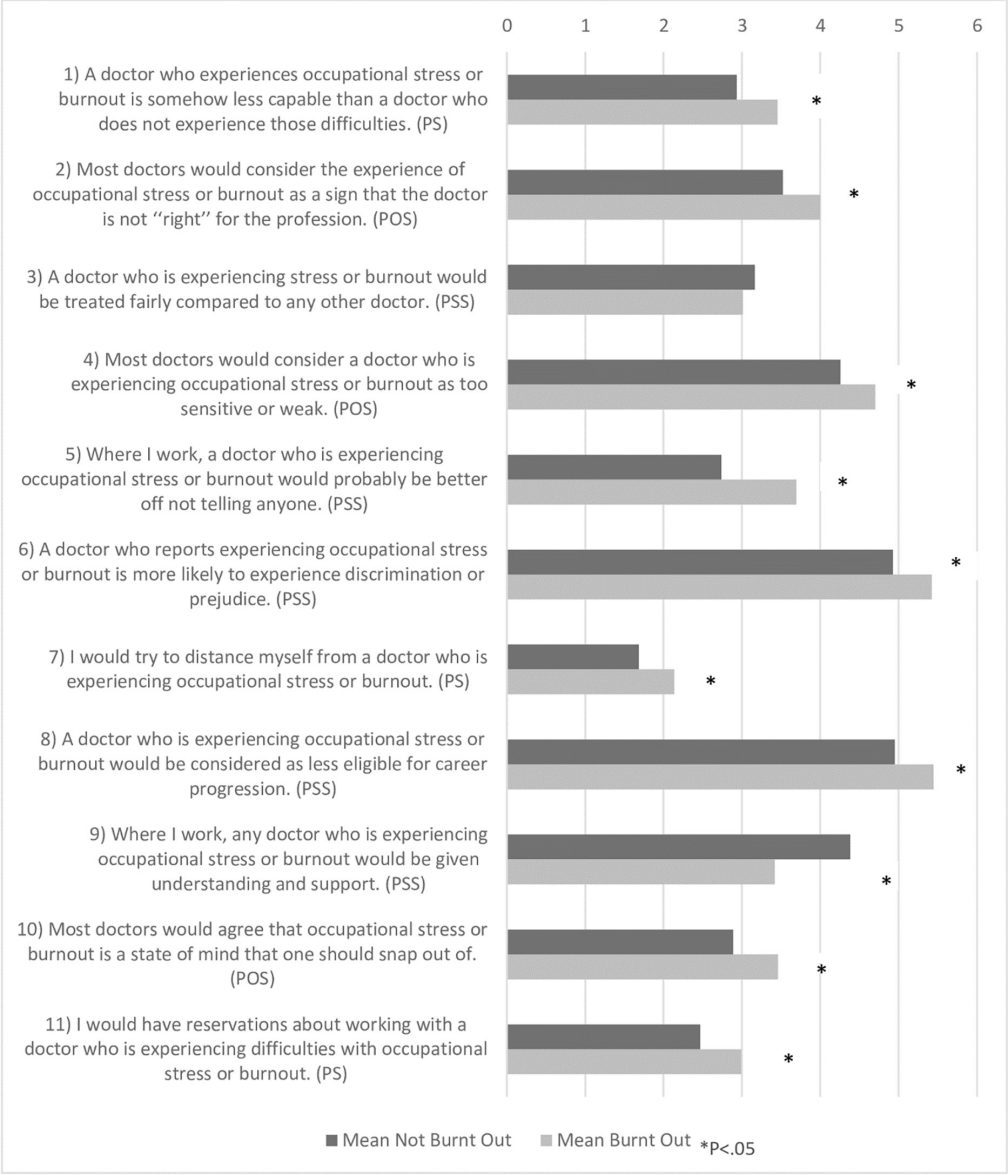
Comparison of stigma by burnout. PS: Personal stigma, POS: Perceived other stigma, and PSS: Perceived structural stigma.

Of the 308 physicians who participated in the study, 163 (53%) did not meet the criteria for burnout. Participants were considered burnt out if both their emotional exhaustion and depersonalization scores were high, while their personal accomplishment scores were low. A comparison of the mean scores of burnout by stigma is presented in Fig 1.

Physicians with burnout were more likely to hold stigmatized views than those without burnout syndrome. Physicians without burnout were more likely to score high on item 9. There was no statistically significant difference in item 3 between the groups. Items 3 and 9 are part of the perceived structural stigma subscale and are both intended to be reverse-scored.

### 3.4 Association between burnout and stigma in physicians

Table 2 shows Pearson’s correlation between each of the stigma dimensions and burnout dimensions. Emotional exhaustion was moderately correlated with perceived structural stigma and weakly associated with perceived other stigma. Depersonalization was weakly related to personal stigma and perceived other stigma. Personal accomplishment was not correlated with any dimension of stigma.

**Table 2:** Associations between subscales of the MBI and subscales of the SOSS-D, presented as Pearson correlation coefficients.

SOSS-D (dependent variable) →	Perceived structural stigma		Personal stigma		Perceived other stigma	
	**Univariate associations**					
**MBI ↓**	Correlation	p	Correlation	p	Correlation	p
**Emotional exhaustion**	0.33	< .001	0.22	< .001	0.25	< .001
**Depersonalization**	0.22	< .001	0.22	< .001	0.24	< .001
**Personal accomplishment**	-0.13	0.02	-0.07	0.23	-0.01	0.83
	**Adjusted associations**					
**MBI ↓**	Correlation	p	Correlation	p	Correlation	p
**Emotional exhaustion**	0.31	<0.001	0.14	0.053	0.18	0.011
**Depersonalization**	0.02	0.71	0.15	0.040	0.17	0.018
**Personal accomplishment**	-0.03	0.65	0.02	0.68	0.10	0.086
	**Adjusted associations, corrected for attenuation**					
**MBI ↓**	Correlation	p	Correlation	p	Correlation	p
**Emotional exhaustion**	0.37	<0.001	0.20	0.053	0.25	0.011
**Depersonalization**	0.03	0.71	0.23	0.040	0.25	0.018
**Personal accomplishment**	-0.04	0.65	0.03	0.68	0.15	0.086

Thus, perceived other stigma and depersonalization were associated as hypothesized. However, the results do not confirm our other hypotheses. Personal stigma was not associated with emotional exhaustion, but with depersonalization. Perceived structural stigma was not associated with personal accomplishment, but with emotional exhaustion.

## 4. Discussion

This study aimed to investigate the potential relationship between burnout dimensions and stigma among physicians working in a university hospital. To the best of our knowledge, this is the first study to address these issues in this context. Overall, our results show a link between stigma and burnout among burnt out physicians. Still, our results do not confirm our assumptions of dimension-by-dimension association: emotional exhaustion was moderately correlated with perceived structural stigma and weakly correlated with perceived other stigma but not with personal stigma. Depersonalization was associated with personal and other perceived stigmas. Personal accomplishment was not associated with any stigma dimensions.

In their review describing the prevalence of burnout among physicians, Rotenstein et al. [2] showed that studies variably reported prevalence estimates of overall burnout or burnout sub-components: 67.0% for general burnout, 72.0% for emotional exhaustion, 68.1% for depersonalization, and 63.2% for low personal accomplishment. A recent review described the prevalence of burnout among general practitioners [41] and noticed that in that population, mean burnout estimates were: 16.43 for emotional exhaustion, which represents a low degree of emotional exhaustion, 6.74 for depersonalization, which represents a moderate degree of this dimension, and a low degree of personal accomplishment (29.28). This heterogeneity in prevalence highlights the importance of considering the work context when considering burnout.

In this study, mean sub-scores for stigma were higher for perceived structural stigma, like in other studies [33], and personal stigma was rated the lowest. These findings show that the perception of the stigma of stress and burnout is mainly situated within the context of the structural influences operating in the workplace. These concerns about the policies and practices that restrict the opportunities of stigmatized persons may prevent physicians from disclosing psychological distress and seeking help.

There was a significant difference in how physicians with burnout syndrome experienced stigma compared with physicians without burnout syndrome. Most items on the stigma scale were higher in those with burnout. Physicians with burnout were likelier to hold stigmatized views as most items had a statistical difference. These results go in the same direction. Namely, high burnout correlates with high stigma and low burnout with lower perceived stigma. Dyrbye et al. [32] assessed the perceptions of stigma among medical students with burnout. They used the MBI to measure burnout and selected items from existing literature on stigma in the field of mental illness. Students with burnout had significantly higher perceived stigma scores and greater fear of discrimination and confidentiality breaches than those without burnout. Similarly, Weiss et al. [42] examined the relationship between burnout and fellows’ perceptions of stigma surrounding help-seeking for mental illness. They concluded that fellows with burnout were more likely than their peers to perceive significant stigma around help-seeking for psychological distress.

Our results show a more complex pattern than the one-to-one association between the different dimensions of burnout and stigma we hypothesized. The primary association was between perceived structural stigma and emotional exhaustion. At the same time, we hypothesized that personal stigma would be associated with emotional exhaustion and that perceived structural stigma would be related to (lack of) personal accomplishment. This dimension of burnout was not associated with any stigma. Our results show that physicians who perceive discrimination and fear of their career and may choose not to disclose their distress (perceived structural stigma) are also more likely to feel tired and unable to face the demands of their job or engage with people (emotional exhaustion). Emotional exhaustion is a core dimension of burnout [32]. In our results, emotional exhaustion was also associated with perceived other stigma and was weakly associated with personal stigma. Perceived other stigma and personal stigma were also associated with another dimension of burnout (depersonalization). We hypothesized that perceived other stigma would only be correlated with depersonalization because of a negative loop. In addition, in the results, the perceived stigma of being less capable and bringing disrepute to the profession (personal stigma) was also linked with a feeling of incapacity to face job demands (emotional exhaustion) and disengagement (depersonalization).

In a recent study [43], nonprofessional occupational mental health staff’s perceived mental illness-related stigma was assessed using Link’s Devaluation-Discrimination Scale [44], and burnout was evaluated using the MBI. These results suggest that higher perceived mental illness-related stigma is associated with more severe burnout. Mental-illness-related stigma significantly affected the MBI depersonalization subscale sore, but not other burnout dimensions. Our study showed that emotional exhaustion is associated with the three factors of perceived stigma and that depersonalization is associated with the SOSS-D subscales related to interpersonal attitudes (personal stigma and perceived other stigma). Perceived stigma at work may also have progressive dimensions. Stigma is a complex and multidimensional phenomenon, and various measures exist. Perceived burnout stigma can also be assessed as a unidimensional construct [45]; they developed an eight-item instrument (BSI-8) that predicts burnout and other mental health indicators, such as depression, anxiety, and stress in adults. They conceptualized burnout stigma as the perceived stigma of individuals experiencing burnout. The differences between the scales measuring stigma might have led to different results. Nevertheless, the results of different studies have shown the same trend. In the future, various approaches, such as qualitative research, could enhance comprehensive results related to the phenomenological experiences of burnout and stigmatization of individuals and their relationship with help-seeking.

The psychological mechanisms underlying the association between stigma and burnout remain unclear. Different models have been previously described. The first model considers stigmatizing attitudes as related to adverse outcomes, which emphasizes the role of the stigmatizer’s psychological distress in the cyclical process of stigma [46]. Burnout stigmatization can also be understood through the lens of social-cognitive stigma theory: people approve of the stereotype that individuals who are burnt out are somehow at fault for their condition and should work harder to meet societal demands [45]. The psychological flexibility model has also been applied to stigma-related problems. Among people suffering from depression, the combination of the increased potential for aversive experiences, a tendency for experiential avoidance, and rigid patterns of behavior under the control of cognitions that are insensitive to the current context are grouped under the term “psychological inflexibility” processes [47]. Burnout has been shown to overlap with depression in terms of etiology, symptoms, course, and cognitive bias [48,49]. Psychological flexibility is negatively correlated with stigma [46] and has a critical indirect role in stigma via emotional exhaustion [50]. Our results fit this flexibility model, as the emotional exhaustion subscale was associated with three factors of perceived stigma. Moreover, in our study, physicians suffering from burnout also had a higher perceived stigma. These characteristics will, in turn, render them unable to empathize with other medical doctors presenting with burnout symptoms. This process can contribute to burnout contagion, given that personal burnout may increase collective burnout, both directly and indirectly [45]. Emotional contagion has been defined as a tendency to converge emotionally by automatically synchronizing facial expressions and postures with those of another person [51]. Several studies have demonstrated that the experience of burnout is contagious and can be transferred directly from one employee to another [52,53] and that the contagion of emotion instigates incivility in the workplace, which in turn is related to two dimensions of burnout (emotional exhaustion and cynicism) [54]. Victims and perpetrators of incivility both suffer from heightened burnout. Indirect burnout can be transferred through the influence of burned-out individuals on the working conditions of others. For example, burnt-out individuals, who have been found to make more mistakes, misuse work breaks, and be more absent from work, can make their co-workers’ jobs more difficult and increase their workload [45]. Our results introduce a new explanation for burnout contagion, as they theoretically show that high burnout and high perceived stigma work together in interpersonal relationships. Physicians with high perceived stigma would not only stigmatize their peers but would also be less empathic, supportive, and understanding. Burnout and stigma increased among colleagues. Burnout and stigma can produce a lack of conversations about burnout, encouragement of support groups, increased vigilance for signs of burnout, and action for suffering colleagues, especially in hierarchical relationships. Further research is required to confirm these results.

The results suggest the need for anti-stigma or anti-burnout interventions for physicians working in hospitals. The reported barriers to treatment and active help-seeking include lack of time, cost, concerns regarding confidentiality, potential career implications, exposure to unwanted interventions, and stigmatization [55–58]. Dyrbye et al. [32] highlighted that perceived stigma could contribute to not seeking help, together with negative personal experiences and the hidden curriculum. Shah et al. [59] advocated for politically significant steps to minimize COVID-19-specific contributing factors for burnout. In Mexico, a program was launched to prevent burnout among physicians and health care professionals. The author explained the lack of attendance in the programs to the “normalization of stress and/or stigma among healthcare professionals” [60]. Bianchi et al. [48] explored senior doctors’ views on mental illness within the medical profession through semi-structured interviews. These findings suggest that more significant efforts are needed to destigmatize mental illness in work and improve support for doctors. Similarly, Martin et al. [61] showed that medical students could benefit from exposure to physicians with self-disclosed histories of coming mental illnesses. Winstanley (2018) [62] developed a web-based intervention to support doctors and medical students in their decision to disclose their mental illness. Walsh et al. [63] explored interventions for the prevention and reduction of work stress and burnout in hospital doctors using a qualitative design a. They concluded that the culture of medicine needs to change from stigmatization and competitiveness to compassion and collaboration. Additionally, promoting psychological flexibility could be a powerful tool for reducing stigmatizing attitudes and burnout [50]. Several interventions have been evaluated for managing physician burnout. Clough et al. [64] conducted a systematic review of psychosocial interventions targeting occupational stress and burnout among doctors. Cognitive behavioral interventions demonstrated the most substantial evidence, particularly for reducing stress. Patel et al. [9] reviewed strategies to manage physician burnout. These include professional coaching and stress reduction programs, team-building activities, paid leave, and time off. Walsh et al. [63] qualitatively explored interventions for the prevention and reduction of work stress and burnout in hospital doctors. They concluded that the culture of medicine needs to change from stigmatization and competitiveness to compassion and collaboration.

This study has several limitations. First, the cross-sectional design of this study allows for the interpretation of correlations rather than causality. Burnout and perceived stigma are interrelated in physicians, and it remains unclear whether burnout generates perceived stigma or conversely. Second, nonresponse bias must be considered, as non-responders may be more exposed to burnout and perceived stigma. Or, on the contrary, it is possible that those more affected by burnout have been more interested in participating. Third, social desirability bias was not controlled. Participants may have answered these sensitive questions with socially desirable rather than truthful responses. Intensive care unit physicians and surgeons were not assessed. Surgical residents have high overall burnout scores [65], and Fu et al. [66] commented on the efforts needed to dismantle the institutionalized stigmatization of mental illness about suicide among surgical trainees. Fourth, the alpha of the three factors of the stigma scale was low. While the sample size was large enough to conduct the analyses, future studies with larger sample sizes should be conducted to provide stronger evidence.». Finally, these results were representative of a single institution. These factors limit the generalizability of the results because there are undoubtedly specialty-related cultures that influenced our results. Future studies should include a more extensive range of specialties to confirm our findings.

To conclude, the results of this study provide a broader understanding of the experience of burnout among medical doctors, which suggests the need for adjustment of existing burnout management at the institutional level. Further research is needed to explore how increased burnout and stigmatization impact collective burnout and stigmatization, as well as treatment delay.

## References

[1] Maslach C. Finding solutions to the problem of burnout. Consult Psychol J. 2017;69(2):143–52. doi: 10.1037/cpb0000090

[2] Rotenstein LS, Torre M, Ramos MA, Rosales RC, Guille C, Sen S, et al. Prevalence of Burnout Among Physicians: A Systematic Review. JAMA. 2018;320(11):1131–50. doi: 10.1001/jama.2018.12777 ; PubMed Central PMCID: PMC6233645. 30326495 PMC6233645

[3] Rodrigues H, Cobucci R, Oliveira A, Cabral JV, Medeiros L, Gurgel K, et al. Burnout syndrome among medical residents: A systematic review and meta-analysis. PLoS One. 2018;13(11):e0206840. Epub 20181112. doi: 10.1371/journal.pone.0206840 ; PubMed Central PMCID: PMC6231624. 30418984 PMC6231624

[4] Shanafelt TD, Boone S, Tan L, Dyrbye LN, Sotile W, Satele D, et al. Burnout and satisfaction with work-life balance among US physicians relative to the general US population. Arch Intern Med. 2012;172(18):1377–85. doi: 10.1001/archinternmed.2012.3199 . 22911330

[5] Spinelli WM. The phantom limb of the triple aim. Mayo Clin Proc. 2013;88(12):1356–7. doi: 10.1016/j.mayocp.2013.08.017 . 24290108

[6] Back AL, Deignan PF, Potter PA. Compassion, compassion fatigue, and burnout: key insights for oncology professionals. Am Soc Clin Oncol Educ Book. 2014:e454–9. doi: 10.14694/EdBook_AM.2014.34.e454 . 24857139

[7] Gleichgerrcht E, Decety J. Empathy in clinical practice: how individual dispositions, gender, and experience moderate empathic concern, burnout, and emotional distress in physicians. PLoS One. 2013;8(4):e61526. Epub 20130419. doi: 10.1371/journal.pone.0061526 ; PubMed Central PMCID: PMC3631218. 23620760 PMC3631218

[8] Kuhn CM, Flanagan EM. Self-care as a professional imperative: physician burnout, depression, and suicide. Can J Anaesth. 2017;64(2):158–68. Epub 20161201. doi: 10.1007/s12630-016-0781-0 . 27910035

[9] Patel RS, Sekhri S, Bhimanadham NN, Imran S, Hossain S. A Review on Strategies to Manage Physician Burnout. Cureus. 2019;11(6):e4805. Epub 20190603. doi: 10.7759/cureus.4805 ; PubMed Central PMCID: PMC6682395. 31404361 PMC6682395

[10] Wallace JE, Lemaire JB, Ghali WA. Physician wellness: a missing quality indicator. Lancet. 2009;374(9702):1714–21. doi: 10.1016/S0140-6736(09)61424-0 . 19914516

[11] Yates SW. Physician Stress and Burnout. Am J Med. 2020;133(2):160–4. Epub 20190911. doi: 10.1016/j.amjmed.2019.08.034 . 31520624

[12] West CP, Shanafelt TD, Kolars JC. Quality of life, burnout, educational debt, and medical knowledge among internal medicine residents. JAMA. 2011;306(9):952–60. doi: 10.1001/jama.2011.1247 . 21900135

[13] Prins JT, Hoekstra-Weebers JE, Gazendam-Donofrio SM, Dillingh GS, Bakker AB, Huisman M, et al. Burnout and engagement among resident doctors in the Netherlands: a national study. Med Educ. 2010;44(3):236–47. doi: 10.1111/j.1365-2923.2009.03590.x . 20444054

[14] Shanafelt TD, Bradley KA, Wipf JE, Back AL. Burnout and self-reported patient care in an internal medicine residency program. Ann Intern Med. 2002;136(5):358–67. doi: 10.7326/0003-4819-136-5-200203050-00008 . 11874308

[15] Sulaiman CFC, Henn P, Smith S, O’Tuathaigh CMP. Burnout syndrome among non-consultant hospital doctors in Ireland: relationship with self-reported patient care. Int J Qual Health Care. 2017;29(5):679–84. doi: 10.1093/intqhc/mzx087 . 28992145

[16] Zhang Y, Feng X. The relationship between job satisfaction, burnout, and turnover intention among physicians from urban state-owned medical institutions in Hubei, China: a cross-sectional study. BMC Health Serv Res. 2011;11:235. Epub 20110924. doi: 10.1186/1472-6963-11-235 ; PubMed Central PMCID: PMC3197494. 21943042 PMC3197494

[17] Chenevert D, Kilroy S, Johnson K, Fournier PL. The determinants of burnout and professional turnover intentions among Canadian physicians: application of the job demands-resources model. BMC Health Serv Res. 2021;21(1):993. Epub 20210920. doi: 10.1186/s12913-021-06981-5 ; PubMed Central PMCID: PMC8454159. 34544396 PMC8454159

[18] Leape LL, Fromson JA. Problem doctors: is there a system-level solution? Ann Intern Med. 2006;144(2):107–15. doi: 10.7326/0003-4819-144-2-200601170-00008 . 16418410

[19] Adeolu JO, Yussuf OB, Popoola OA. Prevalence and Correlates of Job Stress among Junior Doctors in the University College Hospital, Ibadan. Ann Ib Postgrad Med. 2016;14(2):92–8. ; PubMed Central PMCID: PMC5354627. 28337094 PMC5354627

[20] Bernburg M, Vitzthum K, Groneberg DA, Mache S. Physicians’ occupational stress, depressive symptoms and work ability in relation to their working environment: a cross-sectional study of differences among medical residents with various specialties working in German hospitals. BMJ Open. 2016;6(6):e011369. Epub 20160615. doi: 10.1136/bmjopen-2016-011369 ; PubMed Central PMCID: PMC4916614. 27311909 PMC4916614

[21] Clough BA, March S, Leane S, Ireland MJ. What prevents doctors from seeking help for stress and burnout? A mixed-methods investigation among metropolitan and regional-based australian doctors. J Clin Psychol. 2019;75(3):418–32. Epub 20181115. doi: 10.1002/jclp.22707 . 30431644

[22] Balch CM, Shanafelt T. Combating stress and burnout in surgical practice: a review. Adv Surg. 2010;44:29–47. doi: 10.1016/j.yasu.2010.05.018 . 20919512

[23] Ishak WW, Lederer S, Mandili C, Nikravesh R, Seligman L, Vasa M, et al. Burnout during residency training: a literature review. J Grad Med Educ. 2009;1(2):236–42. doi: 10.4300/JGME-D-09-00054.1 ; PubMed Central PMCID: PMC2931238. 21975985 PMC2931238

[24] Cole TR, Carlin N. The suffering of physicians. Lancet. 2009;374(9699):1414–5. doi: 10.1016/s0140-6736(09)61851-1 . 19866520

[25] Gabbard GO. Medicine and its discontents. Mayo Clin Proc. 2013;88(12):1347–9. doi: 10.1016/j.mayocp.2013.10.007 . 24290105

[26] Tijdink JK, Vergouwen AC, Smulders YM. Publication pressure and burn out among Dutch medical professors: a nationwide survey. PLoS One. 2013;8(9):e73381. Epub 20130904. doi: 10.1371/journal.pone.0073381 ; PubMed Central PMCID: PMC3762753. 24023865 PMC3762753

[27] Seidler A, Thinschmidt M, Deckert S, Then F, Hegewald J, Nieuwenhuijsen K, et al. The role of psychosocial working conditions on burnout and its core component emotional exhaustion—a systematic review. J Occup Med Toxicol. 2014;9(1):10. Epub 20140314. doi: 10.1186/1745-6673-9-10 ; PubMed Central PMCID: PMC4233644. 24628839 PMC4233644

[28] Link BG. Stigma: many mechanisms require multifaceted responses. Epidemiol Psichiatr Soc. 2001;10(1):8–11. doi: 10.1017/s1121189x00008484 . 11381480

[29] Cohen D, Winstanley SJ, Greene G. Understanding doctors’ attitudes towards self-disclosure of mental ill health. Occup Med (Lond). 2016;66(5):383–9. Epub 20160329. doi: 10.1093/occmed/kqw024 ; PubMed Central PMCID: PMC4913366. 27030052 PMC4913366

[30] Mehta SS, Edwards ML. Suffering in Silence: Mental Health Stigma and Physicians’ Licensing Fears. Am J Psychiatry Resid J. 2018;13(11):2–4. doi: 10.1176/appi.ajp-rj.2018.131101

[31] Vayr F, Herin F, Jullian B, Soulat JM, Franchitto N. Barriers to seeking help for physicians with substance use disorder: A review. Drug Alcohol Depend. 2019;199:116–21. Epub 20190418. doi: 10.1016/j.drugalcdep.2019.04.004 . 31035230

[32] Dyrbye LN, Eacker A, Durning SJ, Brazeau C, Moutier C, Massie FS, et al. The Impact of Stigma and Personal Experiences on the Help-Seeking Behaviors of Medical Students With Burnout. Acad Med. 2015;90(7):961–9. doi: 10.1097/ACM.0000000000000655 . 25650824

[33] Clough BA, Ireland MJ, March S. Development of the SOSS-D: a scale to measure stigma of occupational stress and burnout in medical doctors. J Ment Health. 2019;28(1):26–33. Epub 20170904. doi: 10.1080/09638237.2017.1370642 . 28868957

[34] Nearchou FA, Bird N, Costello A, Duggan S, Gilroy J, Long R, et al. Personal and perceived public mental-health stigma as predictors of help-seeking intentions in adolescents. J Adolesc. 2018;66:83–90. Epub 20180523. doi: 10.1016/j.adolescence.2018.05.003 . 29800758

[35] Bracke P, Delaruelle K, Verhaeghe M. Dominant Cultural and Personal Stigma Beliefs and the Utilization of Mental Health Services: A Cross-National Comparison. Front Sociol. 2019;4:40. Epub 20190508. doi: 10.3389/fsoc.2019.00040 ; PubMed Central PMCID: PMC8022809. 33869363 PMC8022809

[36] Cho HL, Huang CJ. Why Mental Health-Related Stigma Matters for Physician Wellbeing, Burnout, and Patient Care. J Gen Intern Med. 2020;35(5):1579–81. Epub 20200224. doi: 10.1007/s11606-019-05173-6 ; PubMed Central PMCID: PMC7210325. 32096078 PMC7210325

[37] Maslach C, Jackson SE. The measurement of experienced burnout. J Organ Behav. 1981;2(2):99–113. doi: 10.1002/job.4030020205

[38] Dion G, Tessier R. Validation de la traduction de l’Inventaire d’épuisement professionnel de Maslach et Jackson. [Validation of a French translation of the Maslach Burnout Inventory (MBI).]. Can J Behav Sci. 1994;26(2):210–27. doi: 10.1037/0008-400X.26.2.210

[39] Maslach C, Schaufeli WB, Leiter MP. Job burnout. Annu Rev Psychol. 2001;52:397–422. doi: 10.1146/annurev.psych.52.1.397 . 11148311

[40] StataCorp. Stata Statistical Software: Release 16. College Station, TX: StataCorp LLC.; 2019.

[41] Karuna C, Palmer V, Scott A, Gunn J. Prevalence of burnout among GPs: a systematic review and meta-analysis. Br J Gen Pract. 2022;72(718):e316–e24. Epub 20220428. doi: 10.3399/BJGP.2021.0441 ; PubMed Central PMCID: PMC8869191. 34990391 PMC8869191

[42] Weiss AK, Quinn SM, Danley AL, Wiens KJ, Mehta JJ. Burnout and Perceptions of Stigma and Help-Seeking Behavior Among Pediatric Fellows. Pediatrics. 2021;148(4). Epub 20210924. doi: 10.1542/peds.2021-050393 . 34561267

[43] Mitake T, Iwasaki S, Deguchi Y, Nitta T, Nogi Y, Kadowaki A, et al. Relationship between Burnout and Mental-Illness-Related Stigma among Nonprofessional Occupational Mental Health Staff. Biomed Res Int. 2019;2019:5921703. Epub 20190924. doi: 10.1155/2019/5921703 ; PubMed Central PMCID: PMC6778926. 31662983 PMC6778926

[44] Link BG. Understanding labeling effects in the area of mental disorders: An assessment of the effects of expectations of rejection. Am Sociol Rev. 1987;52(1):96–112. doi: 10.2307/2095395

[45] May RW, Terman JM, Foster G, Seibert GS, Fincham FD. Burnout Stigma Inventory: Initial Development and Validation in Industry and Academia. Front Psychol. 2020;11:391. Epub 20200312. doi: 10.3389/fpsyg.2020.00391 ; PubMed Central PMCID: PMC7080824. 32226404 PMC7080824

[46] Masuda A, Price M, Anderson PL, Schmertz SK, Calamaras MR. The role of psychological flexibility in mental health stigma and psychological distress for the stigmatizer. J Soc Clin Psychol. 2009;28:1244–62. doi: 10.1521/jscp.2009.28.10.1244

[47] Twohig MP, Levin ME. Acceptance and Commitment Therapy as a Treatment for Anxiety and Depression: A Review. Psychiatr Clin North Am. 2017;40(4):751–70. doi: 10.1016/j.psc.2017.08.009 . 29080598

[48] Bianchi R, Schonfeld IS. Burnout is associated with a depressive cognitive style. Pers Individ Dif. 2016;100:1–5. doi: 10.1016/j.paid.2016.01.008

[49] Bianchi R, Schonfeld IS, Laurent E. Burnout Syndrome and Depression. In: Kim Y-K, editor. Understanding Depression: Volume 2 Clinical Manifestations, Diagnosis and Treatment. Singapore: Springer Singapore; 2018. p. 187–202.

[50] Yavuz KF, Nalbant A, Ulusoy S, Esen B, Burhan HS, Kara T. Burned out and Avoided: Stigmatizing Processes among Psychiarists. Psychiatr Danub. 2020;32(Suppl 4):463–70. . 33212450

[51] Hatfield E, Cacioppo JT, Rapson RL. Emotional contagion. New York, NY, US: Cambridge University Press; Paris, France: Editions de la Maison des Sciences de l’Homme; 1994. vii, 240–vii, p.

[52] Bakker AB, van Emmerik H, Euwema MC. Crossover of Burnout and Engagement in Work Teams. Work Occup. 2006;33:464–89. doi: 10.1177/0730888406291310

[53] Bakker AB, Westman M, Schaufeli WB. Crossover of burnout: An experimental design. Eur J Work Organ Psychol. 2007;16:220–39. doi: 10.1080/13594320701218288

[54] Petitta L, Jiang L. Burning out? Watch your own incivility and the emotions you spread. Work. 2019;64(4):671–83. doi: 10.3233/WOR-193029 . 31815707

[55] Brower KJ. Professional Stigma of Mental Health Issues: Physicians Are Both the Cause and Solution. Acad Med. 2021;96(5):635–40. doi: 10.1097/ACM.0000000000003998 ; PubMed Central PMCID: PMC8078109. 33885412 PMC8078109

[56] Givens JL, Tjia J. Depressed medical students’ use of mental health services and barriers to use. Acad Med. 2002;77(9):918–21. doi: 10.1097/00001888-200209000-00024 . 12228091

[57] Schnyder N, Panczak R, Groth N, Schultze-Lutter F. Association between mental health-related stigma and active help-seeking: systematic review and meta-analysis. Br J Psychiatry. 2017;210(4):261–8. Epub 20170202. doi: 10.1192/bjp.bp.116.189464 . 28153928

[58] Veal CT. We Burn Out, We Break, We Die: Medical Schools Must Change Their Culture to Preserve Medical Student Mental Health. Acad Med. 2021;96(5):629–31. doi: 10.1097/ACM.0000000000003991 . 33570856

[59] Shah K, Chaudhari G, Kamrai D, Lail A, Patel RS. How Essential Is to Focus on Physician’s Health and Burnout in Coronavirus (COVID-19) Pandemic? Cureus. 2020;12(4):e7538. Epub 20200404. doi: 10.7759/cureus.7538 ; PubMed Central PMCID: PMC7198080. 32377486 PMC7198080

[60] Ng B. Solutions to prevent and address physician burnout during the pandemic in Mexico. Indian J Psychiatry. 2020;62(Suppl 3):S467–S9. Epub 20200928. doi: 10.4103/psychiatry.IndianJPsychiatry_840_20 ; PubMed Central PMCID: PMC7659778. 33227061 PMC7659778

[61] Martin A, Chilton J, Gothelf D, Amsalem D. Physician Self-disclosure of Lived Experience Improves Mental Health Attitudes Among Medical Students: A Randomized Study. J Med Educ Curric Dev. 2020;7:2382120519889352. Epub 20200108. doi: 10.1177/2382120519889352 ; PubMed Central PMCID: PMC7180952. 32363235 PMC7180952

[62] Winstanley SJ. The development of an intervention to support doctors and medical students in their decision whether to disclose their mental ill health. Cardiff: Cardiff University; 2018.

[63] Walsh G, Hayes B, Freeney Y, McArdle S. Doctor, how can we help you? Qualitative interview study to identify key interventions to target burnout in hospital doctors. BMJ Open. 2019;9(9):e030209. Epub 20190905. doi: 10.1136/bmjopen-2019-030209 ; PubMed Central PMCID: PMC6731950. 31492785 PMC6731950

[64] Clough BA, March S, Chan RJ, Casey LM, Phillips R, Ireland MJ. Psychosocial interventions for managing occupational stress and burnout among medical doctors: a systematic review. Syst Rev. 2017;6(1):144. Epub 20170717. doi: 10.1186/s13643-017-0526-3 ; PubMed Central PMCID: PMC5514490. 28716112 PMC5514490

[65] Burhamah W, AlKhayyat A, Oroszlanyova M, Jafar H, AlKhayat A, Alabbad J. The predictors of depression and burnout among surgical residents: A cross-sectional study from Kuwait. Ann Med Surg (Lond). 2021;65:102337. Epub 20210421. doi: 10.1016/j.amsu.2021.102337 ; PubMed Central PMCID: PMC8093889. 33996067 PMC8093889

[66] Fu WW, Gauger PG, Newman EA. Mental Illness and Stigma in Surgical Residencies-An Unspoken Truth. JAMA Surg. 2021;156(2):117–8. doi: 10.1001/jamasurg.2020.2965 . 33052384

